# Monitoring T-Cell Responses in Translational Studies: Optimization of Dye-Based Proliferation Assay for Evaluation of Antigen-Specific Responses

**DOI:** 10.3389/fimmu.2017.01870

**Published:** 2017-12-21

**Authors:** Anja Ten Brinke, Natalia Marek-Trzonkowska, Maria J. Mansilla, Annelies W. Turksma, Karolina Piekarska, Dorota Iwaszkiewicz-Grześ, Laura Passerini, Grazia Locafaro, Joan Puñet-Ortiz, S. Marieke van Ham, Maria P. Hernandez-Fuentes, Eva M. Martínez-Cáceres, Silvia Gregori

**Affiliations:** ^1^Department of Immunopathology, Sanquin Research, Amsterdam, Netherlands; ^2^Landsteiner Laboratory, Academic Medical Centre, University of Amsterdam, Amsterdam, Netherlands; ^3^Laboratory of Immunoregulation and Cellular Therapies, Department of Family Medicine, Medical University of Gdańsk, Gdańsk, Poland; ^4^Immunology Division, Department of Cellular Biology, Germans Trias i Pujol University Hospital and Research Institute, Physiology, and Immunology, Universitat Autònoma Barcelona, Barcelona, Spain; ^5^Department of Clinical Immunology and Transplantology, Medical University of Gdańsk, Gdańsk, Poland; ^6^San Raffaele Telethon Institute for Gene Therapy (SR-Tiget), Division of Regenerative Medicine, Stem Cells and Gene Therapy, IRCCS San Raffaele Scientific Institute, Milan, Italy; ^7^MRC Centre for Transplantation, King’s College London, London, United Kingdom

**Keywords:** tolerance, monitoring, proliferation, antigen-specific, T cells, transplantation, autoimmune diseases, immune-therapies

## Abstract

Adoptive therapy with regulatory T cells or tolerance-inducing antigen (Ag)-presenting cells is innovative and promising therapeutic approach to control undesired and harmful activation of the immune system, as observed in autoimmune diseases, solid organ and bone marrow transplantation. One of the critical issues to elucidate the mechanisms responsible for success or failure of these therapies and define the specificity of the therapy is the evaluation of the Ag-specific T-cell responses. Several efforts have been made to develop suitable and reproducible assays. Here, we focus on dye-based proliferation assays. We highlight with practical examples the fundamental issues to take into consideration for implementation of an effective and sensitive dye-based proliferation assay to monitor Ag-specific responses in patients. The most critical points were used to design a road map to set up and analyze the optimal assay to assess Ag-specific T-cell responses in patients undergoing different treatments. This is the first step to optimize monitoring of tolerance induction, allowing comparison of outcomes of different clinical studies. The road map can also be applied to other therapeutic interventions, not limited to tolerance induction therapies, in which Ag-specific T-cell responses are relevant such as vaccination approaches and cancer immunotherapy.

## Introduction

The induction of antigen (Ag)-specific tolerance in transplanted or autoimmune disease patients is a pre-eminent goal in precision medicine. Progressively, several tolerance-inducing strategies are entering the clinical arena with immune-modulatory drugs, including novel therapeutic antibodies (1, 2) and cell therapies with regulatory T cells (Tregs) or tolerogenic Ag-presenting cells (tolAPCs). Hence, the need for *in vitro* assays to evaluate the immunological mechanisms responsible for failure or success of these therapies is becoming critical. It may discriminate Ag-specific tolerance induction from general immune suppression and potential loss of pathogen-specific immunity. In addition, assessment of Ag-specific memory for tolerance may allow identification of patients in whom tapering of immunosuppression is likely to be safe, thus minimizing risks of adverse effects resulting from the ongoing treatments.

To evaluate Ag-specific responses *in vitro*, peripheral blood mononuclear cells (PBMCs) are the most widely used cells due to their relative convenient accessibility. Various methods to monitor Ag-specific responses have been developed, including measurement of cytokine production of Ag-responding T cells with enzyme-linked immunosorbent assay (ELISA) or enzyme-linked immunospots (ELISpots), or analysis of T-cell proliferation based on ^3^H-thymidine incorporation. Technical developments in the field of flow cytometry opened new possibilities for analysis and characterization of cell sub-populations and their Ag-specific responses using fluorescent dye dilution (3, 4) and flow cytometric assay of specific cell-mediated immune response in activated whole blood (FASCIA) (5, 6). Cell permeant dyes, such as carboxyfluorescein diacetate succinimidyl ester (CFSE), cell trace violet (CTV), and violet proliferation dye 450 (VPD-450), enabled more specific analysis of cell proliferation over several days. Since the dyes are divided equally between daughter cells (7), the number of cell divisions of the proliferating cells can be visualized, thus allowing the theoretical enumeration of Ag-specific cells. Moreover, dividing cells can phenotypically be characterized using antibodies specific for surface markers and/or intracellular cytokines (8). This multiplies the information to be obtained from a single functional assay. It still remains to be defined, however, whether analysis with proliferation dyes is sensitive enough to evaluate the induction of tolerance in transplantation settings and autoimmunity, where the numbers of autoAg-specific cells are generally very low (9, 10).

Only recently, the use of dye proliferation to monitor Ag-specific T-cell responses has been introduced in clinical practice. Responsiveness to insulin in a small number of children in randomized clinical study, Pre-POINT study, demonstrated the value of combining proliferation dye with analysis for specific T helper profiles. The analysis demonstrated that the observed insulin- and pro-insulin-specific proliferating CD4^+^ T cells acquired a Treg phenotype (11). Similarly, Ag-specific T-cell proliferation in response to Derp1 in small cohort of patients undergoing dust mite allergen-specific immunotherapy was used to demonstrate the ability of the treatment to promote unresponsiveness in allergen-specific T helper cells (12). These studies highlight the limitation of applying dye proliferation assay, as T-cell responses could only be evaluated in a fraction of treated patients. However, these examples also indicate that dye proliferation assay can be a valuable tool to better dissect the effect of a given therapy, since in combination with gene profile or phenotypical analysis (i.e., FOXP3 expression or intracytoplasmic staining for cytokines) can help to grasp the mechanism underlying tolerance induction.

Studies correlating transplant outcome with *in vitro* functional studies have been mostly non-conclusive [as reviewed in Ref. (13)]. In a trial of allo-specific tolerance induction, however, an absence of proliferation to the donor was observed in those patients that could continue with immunosuppression withdrawal (14). Besides in a study focused on finding a biomarker signature to detect renal transplant tolerance in humans the comparison of *in vitro* T-cell function between spontaneously tolerant kidney transplant recipients and non-tolerant recipients demonstrated that the best correlation to the clinical status was obtained with donor-specific IFNγ-ELISpot assays (15).

With the aim to join forces in development and implementation of tolerance-inducing cell products, such as Tregs and tolAPCs, a European network action to focus and accelerate cell-based tolerance-inducing therapies (A FACTT, www.afactt.eu) was initiated in 2014 under the umbrella of European Cooperation in Science and Technology (COST). By creating a forum for the exchange and integration of knowledge and expertise, A FACTT aims to minimize overlap and maximize comparison of the diverse tolerance-inducing cell products, but also to create consensus on monitoring parameters, immune-monitoring assays and establish minimum information models (16–18). Therefore, within A FACTT, we have determined the critical steps of a dye-based proliferation assay to monitor Ag-specific T-cell responses useful for assessing the results of tolerance-inducting therapies, since assay harmonization to monitor tolerance induction is essential to compare outcomes of different clinical studies.

In the current study, we propose a road map for the execution and analysis of dye-based proliferation assays for high-sensitivity monitoring of T-cell responses specific for alloAgs, pathogen-derived exogenous Ags, and autoAgs. This approach will be of pivotal importance for defining effects of tolerance-inducing strategies for transplantation and autoimmune diseases.

## Materials and Equipment

### Subjects

Human peripheral blood was obtained from healthy donors upon informed consent in accordance with local ethical committee approval and with the Declaration of Helsinki.

Human peripheral blood was obtained from four multiple sclerosis (MS) patients from the Multiple Sclerosis Unit, Germans Trias I Pujol University Hospital (Badalona, Spain) upon informed consent in accordance with local ethical committee approval and with the Declaration of Helsinki. No patient had clinical exacerbations or was receiving corticosteroid or disease modifying treatments at the moment of the sample collection.

### Cell Preparations

Peripheral blood mononuclear cells were isolated from buffy coats obtained from healthy volunteer blood donor by Ficoll-Uropoline, Ficoll-Hypaque, or Lymphoprep gradient centrifugation and were either used fresh or after storing them in liquid nitrogen. CD4^+^ T cells were separated by negative selection (StemCell Technologies or Miltenyi Biotec) according to manufacturer’s instructions, with a resulting purity of >95%. Dendritic cells and CD3-depleted PBMC were prepared as previously described (19, 20).

### Dye-Labeling and Proliferation

#### Polyclonal Stimulation

Responder cells were washed twice with warm (37°C) PBS to remove serum that affects staining. Then, cells were suspended in warm (37°C) PBS at a concentration of 5 × 10^6^ cells/ml and labeled with various concentrations of CFSE (Invitrogen, USA) or VPD-450 (BD Biosciences, USA) at 37°C for 15 min. Each 5 min cells were vortexed to provide uniform staining. Subsequently, cells were washed with warm (37°C) PBS and then with culture medium (X-VIVO 20; Lonza) supplemented with 10% fetal bovine serum (FBS) and antibiotics pen/strep. After this step, cells were suspended in fresh medium (X-VIVO 20, 10% FBS, pen/strep) and incubated for 24 h. After this time, the labeled cells were collected, washed with fresh medium, counted, seeded on 96-well plates (1 × 10^5^ cells/well), and stimulated with magnetic beads coated with anti-CD3 and anti-CD28 antibodies (Invitrogen) in 1:1 cell:bead ratio. In parallel, stained and not stimulated cells, as well as unstained and stimulated cells, were seeded in the same concentration as controls. After 96 h, cells were collected and stained with 7-AAD (20 min, at RT). Sample of unlabeled and stimulated cells was stained with anti-CD45 V450 mAb (BD Horizon, USA) or with anti-CD45-FITC mAb (BD Biosciences). After viability check, one well of CSFE or VPD-450 stained stimulated cells was mixed with one well of stained unstimulated cells and one well of unstained stimulated cells labeled with anti-CD45 V450 mAb or with anti-CD45 FITC mAb when CFSE or VPD-450 was used, respectively. Immediately after this step, cells were analyzed with flow cytometer (LSRFortessa; BD Biosciences).

#### Staphylococcal Enterotoxin B (SEB) Stimulation

Thawed PBMCs (10 × 10^6^ cells/ml) were incubated with 2 µM of VPD-450 (BD Biosciences) for 7 min at RT in the dark. Afterward, cells were washed twice with medium and resuspended in IMDM 5% human serum (HS) (Sanquin), pen/strep at a concentration of 1 × 10^6^ cells/ml, plated in 24-well plate, and stimulated with 1 µg/ml SEB (Sigma Aldrich). After 4 or 5 days, cells were harvested, washed with PBS, and stained with near-IR dead cell stain (Thermo Fischer Scientific Inc.) for 30 min at RT in the dark. Subsequently, cells were stained with anti-CD3-BUV496, anti-CD4-Ag-presenting cell (APC), and anti-CD8-BUV805 (BD Biosciences, USA) and analyzed with flow cytometer (LSRFortessa; BD Biosciences). Data were analyzed using the FlowJo software (V10).

#### AlloAg and Pathogen-Specific Ag Stimulation

Fresh PBMCs and purified CD4^+^ T cells were labeled eFluor^®^ 670 (10 µM) (eBioscience) and incubated for 10 min at 37°C in the dark. The labeling of cells was stopped by adding 4–5 volumes of cold FBS (Lonza) and incubating the cells on ice for 5 min. Then, cells were washed and resuspended in culture medium: X-VIVO15 medium with 5% HS (BioWhittaker-Lonza), pen/strep (BioWhittaker). To evaluate the allo-specific proliferative response, labeled PBMCs or CD4^+^ T cells were used as responder cells (R, 10^5^ cells/well). As stimulators (S), either autologous (auto)/allogeneic (allo) CD3-depleted PBMCs (APC) at [R:S] ratio of [1:1] or auto/allo mature dendritic cell (mDC) at a [10:1] ratio were used. Cells were cultured for 4–6 days in 200 µl of X-vivo 5% HS in 96-well round-bottom plates. To evaluate the Ag-specific proliferative response labeled PBMCs (2 × 10^5^ cells/well) were plated in 96-well flat-bottom plates and stimulated either with heat-inactivated *Candida albicans* spores (5 × 10^6^ spores/well, kindly provided by L. Romani, University of Perugia) or with tetanus toxoid (5 µg/ml; Enzo Life Sciences), or with total protein extract from a cell line infected with *Varicella zoster* Virus (2.5 µg/ml; Advanced Biotech) in a final volume of 200 µl of X-vivo (BioWhittaker-Lonza) 5% HS. For live/dead cell discrimination, PBMC or CD4^+^ T cells were stained with Pacific Blue™ Succinimidyl Ester (ThermoFisher) at a final concentration of 0.1 µg/ml, according to manufacturer’s instructions. Proliferated cells were counterstained with anti-CD3 Pacific Orange (clone UCHT1), anti-CD4 Pecy7 (clone SK3, BD Bioscience), and anti-CD8 APC-Cy7 (clone SK1, BD) mAbs by 15 min incubation at RT in PBS 2% FBS. Cells were washed with PBS 2% FBS and fixed with 0.25% formaldehyde. Flow cytometry analyses were performed with FCS Express 4 [*De Novo* Software; (https://www.denovosoftware.com/site/manual/proliferation_statistics.htm)], and the frequency of precursors was calculated according to the automatic proliferation fit statistics, as described in the manufacturer’s instructions. Alternatively, after 3, 4, or 5 days, cells were pulsed for 16 h with 1 μCi/well ^3^H-thymidine.

#### AutoAg Stimulation

Fresh PBMCs were labeled with VPD-450 (BD Horizon). A total of 8 × 10^6^ cells/ml were stained with 1 µM VPD-450, 14 min at 37°C, in dark. After two washing steps with PBS, cells were resuspended in 1 ml of RPMI (Sigma-Aldrich) supplemented with 10% FBS, pen/strep (Cepa and Normon, respectively) and 2 mM l-glutamine (Sigma-Aldrich). A total of 1.5 × 10^5^ PBMCs in 200 µl/well (5 wells/patient) were cultured in 96-well round-bottom plates for 7 days at 37°C in the presence of 5 µM of 7 myelin peptides [myelin oligodendrocyte glycoprotein (MOG) 1–20, MOG35–55, PLP139–154, myelin basic protein (MBP) 13–32, MBP83–99, MBP111–129 and MBP146–170]. Non-stimulated PBMC and 25 ng/ml phorbol 12-myristate-13-acetate (PMA) plus 250 ng/ml Ionomycin (Sigma-Aldrich) stimulated blood sample were used as negative and positive control, respectively. After 7 days of culture, PBMCs were stained with CD3-V500 (BD Horizon), CD45-APC, 7-AAD (actinomycin D), and CD4-FITC/CD8-PE (BD Biosciences), acquired with FACS Canto II (BD Bioscience) and analyzed using the FlowJo software. Alternatively, to analyze cell proliferation using ^3^H-thymidine incorporation, after 7 days of cell culture (1.5 × 10^5^ PBMCs/well, 60 wells/patient) with myelin peptides, cells were pulsed for 18 h with 1 μCi/well ^3^H-thymidine.

### Ovalbumin (OVA) Stimulation for Frequency Calculation

#### Responder Cells

BALB/C mice were purchased from Harlan (UK) and DO11.10 naive mice were bred in house and maintained in pathogen-free facilities (mice care was in accordance with institutional guidelines). Naive CD4+ T cells were isolated from splenocytes and peripheral lymph node lymphocytes by incubation with MoAbs: CD8 (56-3.72) and MHC class II (MS/114.15-2), locally produced hybridomas, CD16/32 and B220 (Becton Dickinson BD-Pharmingen), and CD25 (BD-Pharmingen), followed by negative selection using magnetic beads coated with sheep-anti rat IgG antibody (Dynal). Efficacy of depletion was measured by flow cytometry, and in all cases, CD4+ fractions were >85% pure; for DO11.10, the MoAb KJ126 (Caltag) was used. Cell calculations in our population were adjusted using this percentage.

#### Stimulator Cells

CHO cells doubly transfected with mouse CD86 and H-2A^d^ were used as stimulators. CHO cells were maintained in culture as previously described (21), and prior to culture, they were incubated with 30 µg/ml Mitomycin C (Kiowa) for 1 h at 37°C, extensively washed and irradiated at 100 Gy. These cells were used to present OVA peptide 323–339 (Sigma) in the context of H-2Ad. All experiments with murine samples were performed in RPMI 1640 (Sigma) supplemented with pen/strep (Gibco), l-Glutamine 2 mM (Gibco), 10 mM HEPES (Gibco), 2β-mercaptoethanol (Gibco), and 10% fetal calf serum (SeraQ).

#### CFSE Labeling for Frequency Calculation

BALB/C and DO11.10 CD4 T cells were labeled independently with CFSE (Molecular Probes, Leiden, The Netherlands) as follows: 2 × 10^7^ cells were incubated with 1 µM of CFSE for 3 min at RT, washed extensively and were left overnight at 37°C 5% CO_2_ in culture medium. Known numbers of DO11.10 cells into BALB/C were mixed as above and incubated for 96 h at 37°C, 5% CO_2_ with stimulator cells and 0.5 µg/ml OVA_323–339_ peptide. Cells incubated in the absence of peptide were used as negative controls. Cells stimulated with 200 pg/ml phorbol dibutyrate (Sigma) and 1 µM Ionomycin (Sigma) were used as positive controls. Before acquisition in the flow cytometer, cells were labeled with mouse CD4-APC (Caltag), the clonotypic marker KJ126-PE (Caltag), and 20 ng/ml of propidium iodide (Sigma), thus enabling gating of the clonotypic receptor-expressing live CD4+ T cells. This way background proliferation of BALB/C cells to CHO stimulators was easily eliminated.

Flow cytometry analysis was performed before the beginning of the culture and after 3 days using Cell Quest and a FACScalibur (BD). Absolute counts of dividing precursors are achieved using Perfect-Count Micrsopheres (Cytognos) as per manufacturer’s instructions. An absolute number of successful proliferative precursors can thus be obtained, by referring this number to the number of seeded cells in the well the frequency is easily calculated. Frequencies are given as 1 in “*n*” number of cells obtained as mean and standard deviation of the duplicated cultures.

#### ELISpot Assay for Frequency Calculation

A commercial set of reagents was used (AID), and manufacturer’s instructions were followed. Spots were enumerated with an ELISpot reader (AID). Plate was prepared as follows: duplicates at five “1/10” dilutions of mixed responder cells (according to the mix prepared to have 10–1,000 DO11.10 in the well) in 100 µl of medium were seeded. Irradiated and mitomycin-treated stimulator cells (50 × 10^4^ in 50 µl) were added to all wells. OVA_323–339_ peptide at a final concentration of 0.5 mg/ml was also added to the necessary wells. Results are given as mean frequency and standard deviation calculated from the five dilutions in the format of 1 in “*n*” number of cells. Background spots of IL-2 production from negative control well (BALB/C cells and CHO stimulators) per dilution were subtracted from experimental IL-2 spots.

### FASCIA

A total of 1 ml of PBS diluted (1/10) whole blood was stimulated with 50 µM of seven myelin peptides for 7 days at 37°C and 5% CO_2_. Non-stimulated and 25 ng/ml PMA plus 250 ng/ml Ionomycin-stimulated blood samples were used as negative and positive controls, respectively. After 7 days of culture, blood cells were stained with anti-CD3-V500 (BD Horizon), anti-CD45-APC, 7-AAD, and CD4-FITC/CD8-PE (BD Biosciences), and after lysis of erythrocytes, samples were acquired with FACS FACSVerse (BD Biosciences) and analyzed using the FACS Diva software (BD Biosciences). Number of proliferating cells was calculated following the protocol and formulas established in the Karolinska University Hospital (5).

#### IFN-γ Production

Allogeneic-Ag-specific responses: labeled PBMCs (10^5^ cells/well) were activated with irradiated (600 rad) auto or allo CD3-depleted PBMCs (APC) (2 × 10^5^ cells/well) at a responder cells:stimulators ratio of 1:1. Alternatively, labeled PBMCs (1 × 10^5^ cells/well) were stimulated with auto or allo mDC (10^4^cells/well) at a responder cells:stimulators ratio of 10:1 for the indicated time points in a final volume of 200 µl of X-VIVO15 medium with 5% HS (BioWhittaker-Lonza) and pen/strep (BioWhittaker) in 96-well round-bottom plates. Supernatants were harvested after 4 and 5 days of culture, and levels of IFN-γ were determined by ELISA according to the manufacturer’s instructions (BD Biosciences).

Pathogenic Ag-specific responses: labeled PBMCs (2 × 10^5^ cells/well) were left inactivated or stimulated with *C. albicans* spores (10^6^/well heat-inactivated spores generously provided by Prof. L. Romani, University of Perugia, Italy) or tetanus toxoid at 5 µg/ml, or in the presence of total protein extract from a cell line infected with *V. zoster* Virus (5 µg/ml) in a final volume of 200 µl of medium (96-well round-bottom plates). Supernatants were harvested after 3, 4, and 5 days of culture and levels of IFN-γ.

### Statistics

Analysis was performed using the GraphPad Prism 5.0 software. The correlation between the different parameters analyzed was evaluated by the non-parametric Spearman’s rank correlation analysis.

## Stepwise Procedures

**Figure 1:**
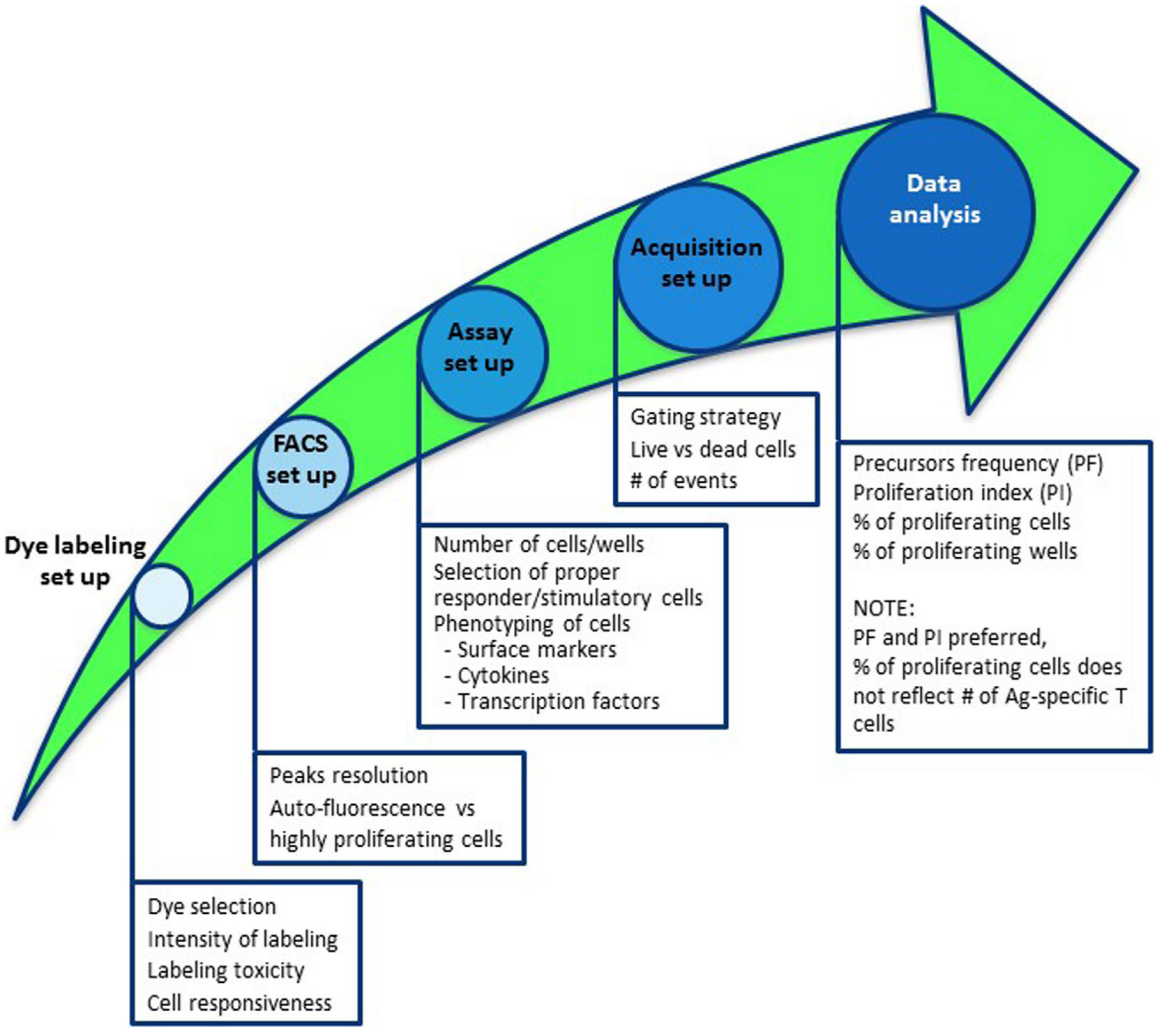
Road map to establish a dye dilution proliferation assay to monitor antigen-specific T-cell responses. Steps to take into consideration to properly set up a dye-based proliferation assay include (1) selection of the appropriate dye and quality control analyses of labeling; (2) defining suitable flow cytometer parameters to perform the analyses; (3) outlining the assay by defining the number of cells/wells to be put in culture, selection of responder and stimulatory cells, duration of culture, including additional staining for comprehensive analyses; (4) sample acquisition by delineating the gating strategy, i.e., live versus dead cells and number of events to acquire; and (5) data analyses.

Functional *in vitro* assays to monitor frequency and phenotype of Ag-specific T-cell responses using fluorescent dye dilution depend on prolonged cell culture and proliferation of Ag-specific cells within the cultures. In these assays, responder cells are labeled by fluorescent dye and upon Ag-specific stimulation the dye is divided equally between daughter cells and the number of cell divisions of the proliferating cells can be visualized, allowing the theoretical enumeration of Ag-specific cells (Figure S1 in Supplementary Material). Here, we outline the critical steps required for establishing and analyzing an appropriate dye-based proliferation assay in a road map (Figure 1). Several technical constrains need to be taken into consideration in the setup of the assay, as they will have a definitive impact on the results of functional *in vitro* assays. Although not the focus of this study, choices of culture medium, serum lot—if used—and storage of reagents are obvious parameters that will affect the results of these assays.

An appropriate culture medium that ensures ample nutrient availability for the cells throughout the whole period of culture should be taken into consideration. Serum remains the preferred source of nutrients; however, most cultures are performed with 5–10% of heat-inactivated fetal calf or bovine serum. If serum is to be added to cultures each lot needs to be tested in all of the stimulations to be used to ensure the best signal-to-noise ratio. We suggest reserving a large amount of the serum lot to acquire consistency across a project. Recently, serum-free media have become popular (22) and ensure consistency of results independent of serum lot. Testing of the above-described variables is beyond the scope of the present work.

### Proliferation Dye Selection: Defining Optimal Dye and Labeling Concentration

Dye-based proliferation assay requires optimization in the laboratory where it will be performed, as for most other cell-based assays. Of specific importance is to optimize PBMC labeling with the dye of choice (23). Different proliferation dyes can be used, i.e., CFSE, CTV, VPD-450, and eFluor^®^ 670. The staining intensity of responder cells should be as high as possible to obtain a broad analysis window of cell division (optimal fluorescence difference between specifically labeled cells and autofluorescenceof unlabeled cells). In this process of optimization, dye toxicity is an important issue to be taken into account. Toxicity of the labeling procedure is essential to be avoided by defining the optimal tolerable dye concentration, which can be monitored through a live/dead staining after culturing of the labeled cells. We recommend not only to focus on the induced toxicity, measurable by live/dead staining, but also to determine the responsiveness of the PBMCs to stimulation as one of the quality controls. Polyclonal stimulation with anti-CD3 and anti-CD28 mAbs can be used to verify the impact of labeling on T-cell proliferation. As an alternative, stimulation with mitogens (PHA and PKW), or superAgs (SEB), can be used.

**Figure 2:**
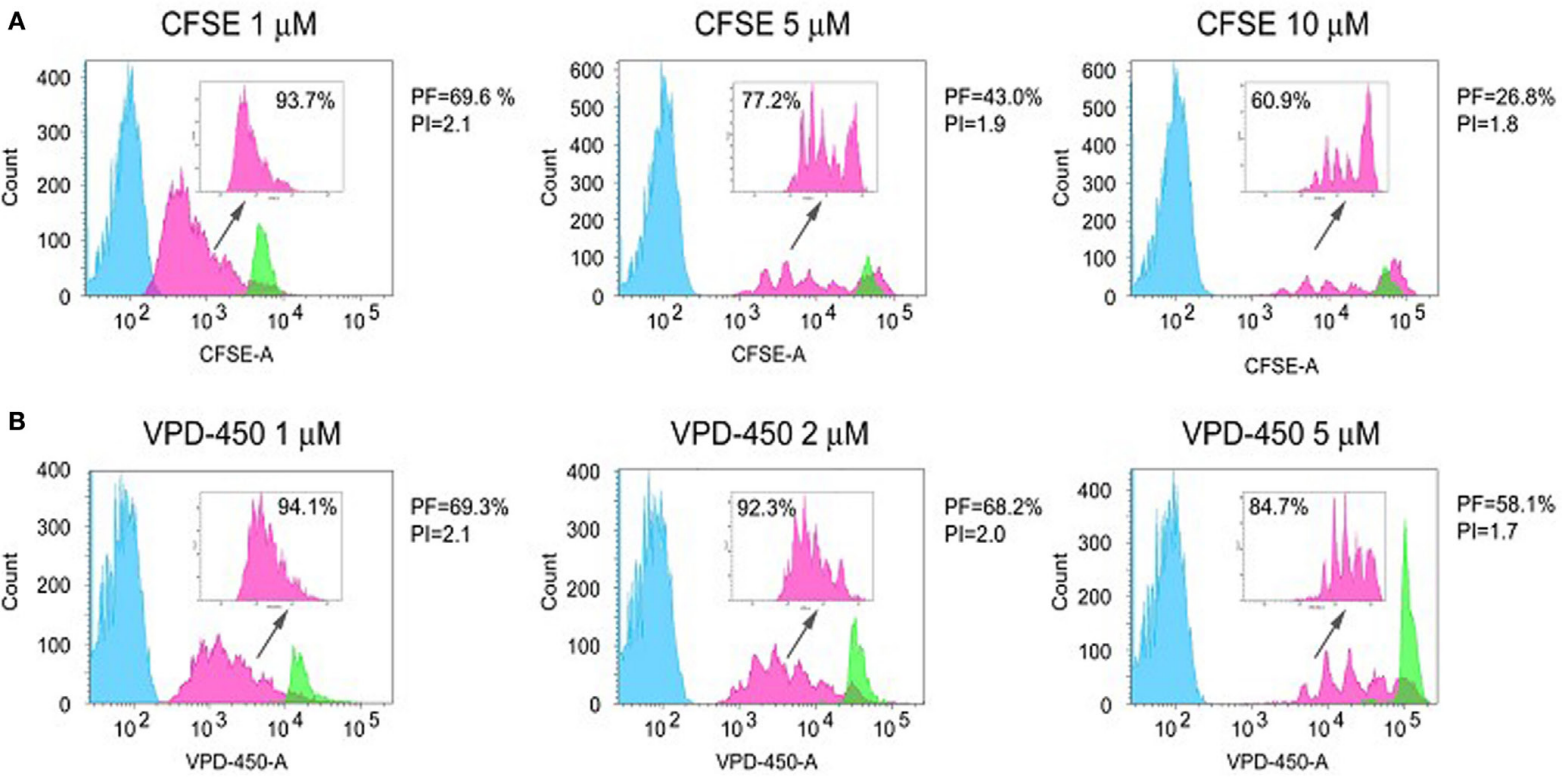
Dye-based proliferation assay: dye selection and optimization of the concentration. Freshly isolated CD4^+^ T cells were labeled with different concentrations of CFSE (1, 5, and 10 µM) **(A)** and VPD-450 (1, 2, and 10 μM) **(B)**, seeded at 1 × 10^5^ cell/well and stimulated with magnetic beads coated with anti-CD3 and anti-CD28 antibodies (cell:bead ratio 1:1) for 4 days. The histograms correspond to unlabeled and stimulated cells (blue, auto-fluorescence), labeled and stimulated cells (pink) and labeled and unstimulated cells (green). The three cell populations were treated, cultured separately, and mixed before the analysis. Prior to the analysis and before mixing the cells, the unstained cells were labeled with anti-CD45 V450 or anti-CD45 FITC antibodies when CFSE or VPD-450 were used, respectively, to detect any overlap between stained and unstained cells. CFSE- and VPD-450-labeled and not stimulated cells were significantly smaller than labeled and stimulated cells; thus, they were discriminated according to the low values of FSC and SSC parameters. In the upper right corner of each histogram, only the proliferation of labeled responders is depicted and % of proliferating cells is presented. For each population only viable (7-AAD^−^), cells are presented. The following parameters are shown: PF = precursor frequency and PI = proliferation index.

An important aspect that should be taken into account during optimization of the labeling procedure is the difference in dye fluorescence intensity between labeled and unlabeled cells, which affects the extent of analysis window. It is therefore clear that the choice of dye and labeling concentration for dye-based proliferation assays should be the result of a clear validation and analysis. In Figure 2, an example of such a dye selection is shown in which 3 concentrations of CFSE (1, 5, and 10 µM) and VPD-450 (1, 2, and 5 µM) were compared to label and analyze isolated total CD4^+^ T cells. After 4-day culture, stimulated labeled cells were harvested and analyzed by flow cytometry, using live/dead staining and optimization of flow cytometer settings for each dye and each dye concentration to allow maximal separation between (auto-fluorescent) unlabeled cells and specifically labeled cells. The use of 5 and 10 µM CFSE led to higher signal intensities (undivided cells reach fifth decade on *X*-axis, green histograms) and better separation of division peaks than 1 µM concentration (Figure 2A). However, CFSE concentrations ≥5 µM were associated with relatively high cell toxicity (25 and 37% dead cells for 5 and 10 µM concentrations, respectively) as compared with 1 µM solution (13% dead cells). In addition, ≥5 µM CFSE concentrations decreased T-cell responsiveness: a lower percentage of responding cells (precursor frequency; PF; pink histograms) was observed for cells stained with 5 and 10 µM CFSE solutions than for 1 µM CFSE concentration. In addition, high dye concentrations decreased the average number of divisions of responding cells (proliferation index; PI). Along the same line, labeling with 5 µM of VPD-450 also led to higher fluorescence intensity than 1 and 2 µM solutions of VPD-450 (Figure 2B). Unlike for CFSE, cells stained with 1, 2 and 5 µM of VPD-450 showed similar viability (13, 18, and 17% of dead cells, respectively), but cell responsiveness (% of proliferating cells, PF and PI) was significantly lower for 5 µM than for 1 and 2 µM concentrations. When results for both dyes were compared, no differences in the dead cell frequency (7-AAD^+^), % of proliferating cells, and PI for 1 µM concentrations were observed. However, staining with 1 µM solution of VPD-450 resulted in a better peak separation than that observed for the same concentration of CFSE. Staining with 2 µM of VPD-450 further improved peak separation (pink histograms) with negligible impact on number of responding cells (PF) and PI. Therefore, for this assay setup, VPD-450 would be chosen as labeling dye at a concentration of 1 or 2 µM. Described analyses underline the importance of a designated assay for the selection of the proliferation dye, as a particular dye and/or its applied concentration may affect not only cell viability but also the proliferative responsiveness of the cells. Toxicity and labeling intensity are influenced by dye concentration, presence or absence of serum or other proteins during the labeling procedure, and the length and the temperature of labeling (24). In general, most laboratories choose to label PBMCs in a protein-free medium, since the used dyes covalently bind to free amines in proteins and hereby labeling of proteins in the medium is prevented. However, Quah and Parish (23) optimized the labeling in a protein rich medium by using high concentrations of dye and described optimal labeling, with low toxicity.

Importantly, every laboratory should perform this selection using their procedures, media, reagents and machines. Thus far, no specific indications regarding the best dye are available and each dye should be carefully tested in each particular setting.

### Peaks Resolution: Autofluorescence versus Cell Proliferation

Proper selection of the dye and its labeling concentration together with dye-optimized flow cytometer setup enable optimal separation of positive signal of responding cells (maximally divided labeled cells) from autofluorescence of the unlabeled cells (Figure 2, blue histograms). Efforts to optimize the signal-to-noise ratio are crucial to distinguish the separate peaks of dividing cells allowing reliable calculation of PFs of the responding cells.

Therefore, to perform optimal proliferation dye-based assays, we recommend to test and validate the most appropriate dye and its concentration with specific flow cytometer setup (Figure 1).

### Assay Setup

#### Definition of the Number of Cells/Wells to Seed

When setting up culture conditions for Ag-specific T-cell enumeration, it is very important to use ≥10-fold more cells per culture than the expected frequency of responders to reliably monitor T-cell responses. Thus, if an Ag-specific response is to be measured in naive human individuals, at least 1 million of the responder cells need to be seeded, as the frequency of many Ag-specific naive T cells is in the order of 1:100,000. In contrast, if a subject has already been exposed to a given Ag, it is likely that 200,000 cells will be enough for detection of a response as the frequency of Ag-specific memory T cells is significantly higher (10, 25–27).

**Figure 3:**
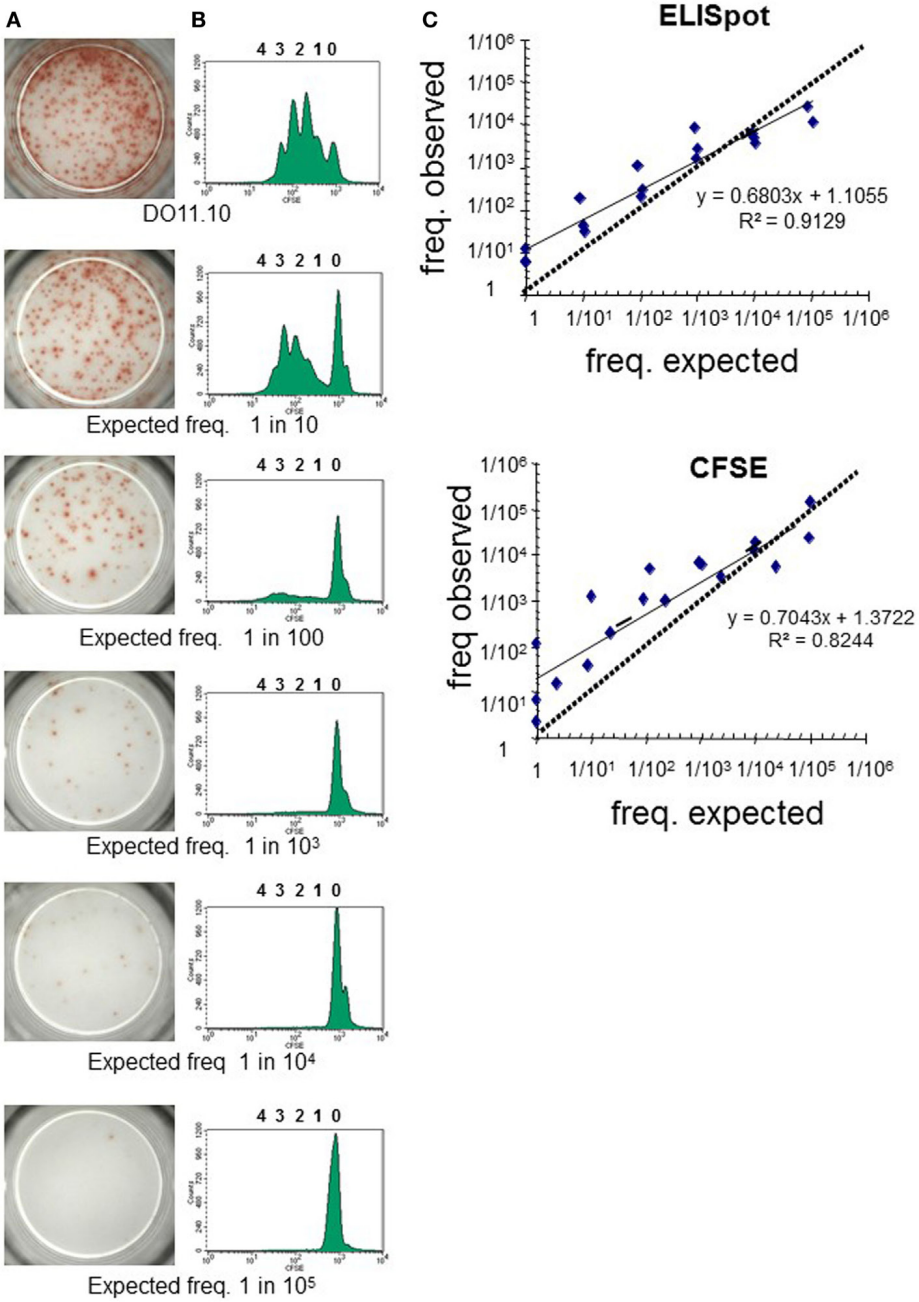
Dye-based T-cell proliferation: sensitivity of assay compared to other approaches. CD4^+^ T cells isolated from the spleen and peripheral lymph nodes of BALB/c and DO11.10 mice were labeled independently with CFSE (1 µM). Known numbers of DO11.10 cells were mixed with BALB/C cells and stimulated with mitomycin C-treated CHO pulsed with OVA_323–339_ peptide for 3 days as in Rogers et al. (21). Experimental responding frequencies were determined by **(A)** IL-2 enzyme-linked immunospot (ELISpot) or **(B)** CFSE dilution. **(A)** Pictures of representative experiment of IL-2 ELISpot wells for each condition tested are shown. **(B)** Representative histograms for each condition tested are depicted. Before acquisition in the flow cytometer cells were labeled with anti-CD4 and anti-KJ126 mAbs, enabling gating of the DO11.10 CD4^+^ T cells. **(C)** Pearson correlation of observed frequencies by ELISpot and CFSE dilution against expected values of three independent experiments are presented. In accurate assays, it would be expected, within a tolerable error, that the measurements fall on a 45° line through the origin.

The threshold of sensitivity of the dye proliferation assay to reliably analyze low frequency T-cell responses is often questioned. The sensitivity of the dye proliferation assay was compared to ELISpot by using mouse TCR-transgenic CD4^+^ T cells specific for OVA (DO11.10 cells) (Figure 3). DO11.10 cells were seeded at different known concentrations together with CD4^+^ T cells from naive BALB/c cells, hereby knowing exactly the expected frequency of Ag-specific T cells to be found in the cultures. T cells were stimulated with OVA peptide-loaded CHO cells that expressed mouse CD86 (21), and the PFs were determined either by IL-2 ELISpot (Figure 3A) or by CFSE proliferation assay (Figure 3B). Results of both assays were in good correlation with the frequency of Ag-specific T cells in the culture (Figure 3C). Notably, at the lower frequencies of Ag-specific T cells (1/10^4^ or 1/10^5^) the measured frequencies were higher than expected, falling under the 45° line. Thus, maybe over-estimating the number of Ag-specific T cells in these settings. Overall, both methods are sensitive enough to reflect differences in frequency of Ag-specific T cells between samples.

When dealing with the low frequency of responder cells, we recommend calculating the number of cells to seed in culture as well as the number of events to be acquired should be calculated according to the estimated frequency of the putative Ag-specific cells present in the culture (4).

#### Proper Selection of the Responder, Stimulator Cells and Optimal Time Point to Monitor Proliferation

An important issue that has to be taken into consideration during optimization of dye-based proliferation assays is the proper selection of the responder and stimulator cells to be used (Figure 1). Responder cells can be either total PBMCs or purified CD4^+^ or CD8^+^ T cells; however, the use of total PBMCs could be theoretically more informative, since it will allow studying the response of different lymphocyte sub-populations (i.e., CD4, CD8, effector, naive, and memory T-cell subsets), and analysis of activation markers (28). Furthermore, (allo)Ag-specific T cells and dye-based proliferation assay can be combined with the intracellular staining for cytokines upon *in vitro* re-stimulation (8, 29, 30) or for transcription factors, such as FOXP3 (31, 32), overall obtaining additional information regarding proportions of different cell subsets, including Tregs within the Ag-specific cell pool present in the culture.

Another important point to take into consideration during optimization of the proliferation dye assay is the selection of the proper time point to visualize effective proliferation and optimal separate division peaks. Examples of how the source of stimulatory cells, the purity of the responder cell population and the timing may impact the *in vitro* detection of alloAg-specific T-cell responses is provided in the Section “Anticipated Results.”

### Acquisition Setup

To analyze proliferation dye data, a gating strategy focusing on living cells and number of acquired events is recommended. Of note, in case, the proliferation dye-based assay is used to analyze alloAg-specific T-cell responses, where allo PBMCs/APCs are added as stimulators to the culture, it is very important to distinguish between proliferation of responder and stimulator cells (both negative for proliferation dye fluorescence). To this end, different approaches can be used including labeling of the responder and stimulator cells with different dyes or depleting CD3^+^ lymphocytes from stimulator PBMCs.

### Optimal Parameters for the Analysis of Proliferation Dye Data

**Figure 4:**
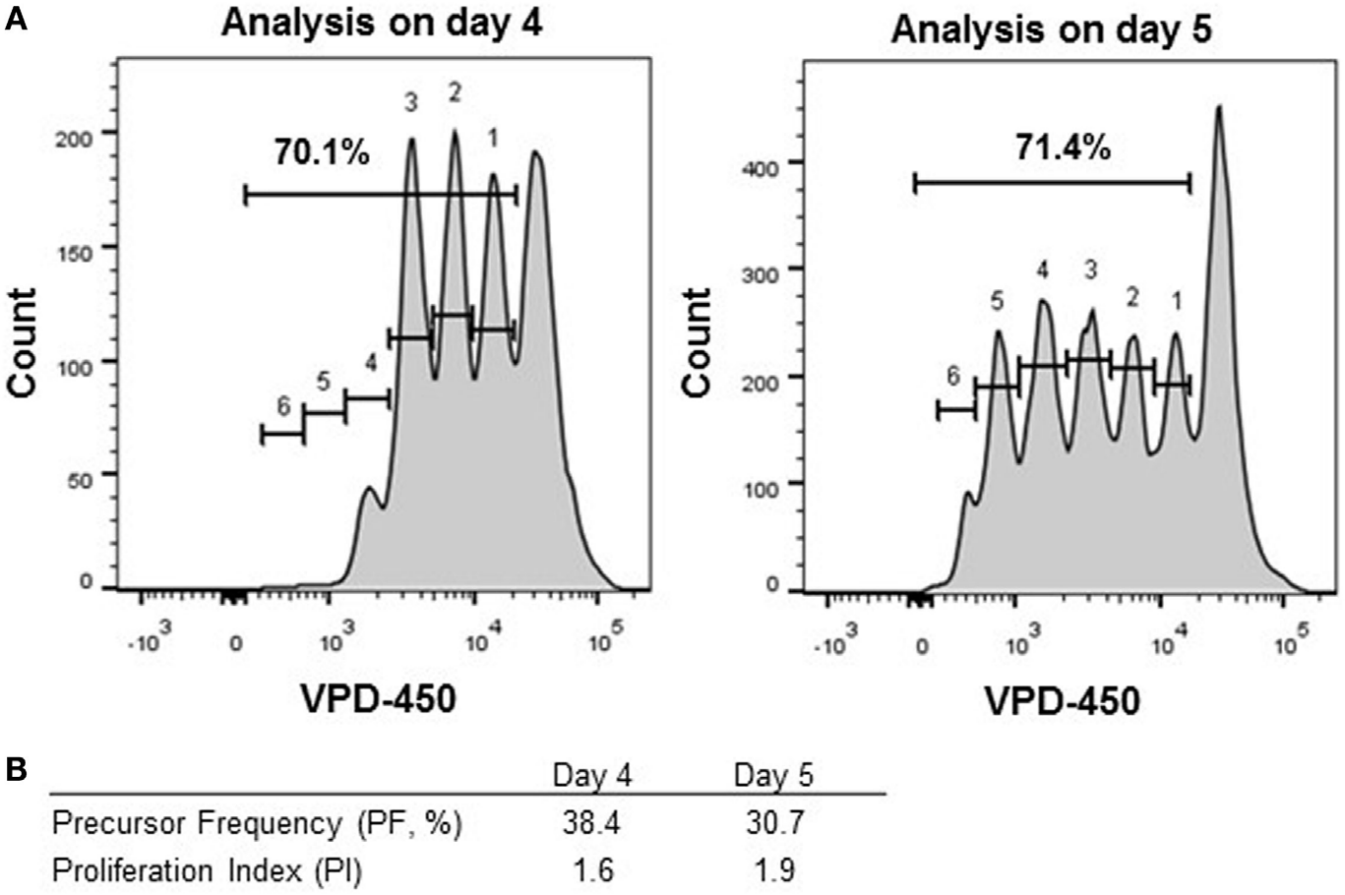
Different modes to analyze proliferating cells. Thawed peripheral blood mononuclear cells were labeled with VPD-450 (2 µM) and stimulated with Staphylococcal enterotoxin B for 4 (left panel) and 5 (right panel) days. **(A)** CD8^+^ T cells were analyzed for the percentage of proliferating cells in total and per division peak. The division peaks are numbered 1 through 6. **(B)** Percentages of proliferating cells, precursor frequency, and the proliferation index have been calculated by using the number of events measured in each division peak.

Results obtained by performing a dye-based proliferation assay can be depicted and interpreted in several manners (Figure 1). Results of proliferation dye assays are generally presented as percentage of cells showing dye dilution (% of proliferating gate, Figure S1 in Supplementary Material). The latter is the easiest and the most often used parameter to present proliferation data. However, this parameter is affected by both the number of cells responding to a given stimulus (PF) and the number of divisions of dividing cells (PI) and, therefore, gives limited insight in the dynamics of cell proliferation and reactive T-cell frequencies. Obviously, this result is affected by several parameters, including actual percentage of cells responsive to stimulus (also named progenitor cells or PF), number of divisions of the dividing cells, and occurrence of cell death. Thus, this parameter is good for general comparison between different samples but is difficult to interpret and may be not sufficient for monitoring Ag-specific responses, since it does not directly reflect real percentage of Ag-specific T cells present in culture. Alternatively, results of proliferation dye can be depicted as (i) PF (proportion of cells with reactivity to a specific Ag or mitogen within the starting population), (ii) division index (DI; average number of divisions of all cells, including undivided cells); and (iii) PI (average number of cell divisions of responding cells). It is recommended to present the PF and PI, since the DI is affected by both the PF and the PI. The calculation of these parameters for each dye-based proliferation culture can be determined by operator or by using flow cytometry analysis software (33, 34). In Figure 4, an example of different ways to depict proliferation dye data is given. Although in both conditions the percentage of proliferating cells was similar (70.1 vs. 71.4%), the different values for the PIs (1.6 vs. 1.9, Table S1 in Supplementary Material) of both cultures showed that the cells had not proliferated to the same extent in the two conditions. The PFs calculated for the two conditions also differed to some extent (PF, 38.4 vs. 30.7%, Table S2 in Supplementary Material).

Together, this example underlines the limitations of analysis of percentage of proliferating cells and concomitantly indicates that the combined use of PI and PF is most informative to analyze results from proliferation dye-based assays for immuno-monitoring. Importantly, percentage of proliferating cells is not an informative parameter when it exceeds 60%, since the ability to distinguish biological variations becomes difficult (34). When monitoring the effect of a tolerizing therapy, depicting both PF and PI separately, when possible, will give more information regarding the mode of tolerance induction. As a drawback, the calculation of PF is not always possible due to the lack of visible separate division peaks. This possibility can occur when autoAg-specific T-cell proliferation is monitored or when alloAg-specific proliferation of cells isolated from patients under immunosuppressive regimens. In the latter case, software peak prediction can be used. Nevertheless, as shown in this study, in some situations also computational prediction cannot be applied. In this case, the only remaining option is to present percentage of proliferating wells, as shown in the present study. Besides, as for all functional assay, it has to be taken into account that the outcomes of the assay will be affected by cell death in the culture, and is a reflection of the surviving cells.

## Anticipated Results (Pitfalls, Artifacts, and Troubleshooting)

To evaluate the mechanisms underlying failure or success of tolerogenic therapies in transplantation or autoimmunity, monitoring of Ag-specific immune responses is critically important. The precise enumeration and phenotypic analysis of Ag-specific T cells remains technically difficult, mainly due to their low frequency (26, 27, 35). Therefore, a sensitive, reproducible, and reliable method to enumerate and analyze Ag-specific T cells in treated subjects is important. Several approaches have been proposed and tested to identify Ag-specific T cells, including proliferative responses and cytokine production profiles. In this study, we focus on the use of T-cell proliferation for detection and analysis of Ag-specific T cells in PBMCs. We provide evidences that a dye-based proliferation assay is as sensitive as other currently used methods for enumerating low frequency Ag-specific T cells.

### Sensitivity of the Dye Proliferation Assay Compared to Other Approaches

**Figure 5:**
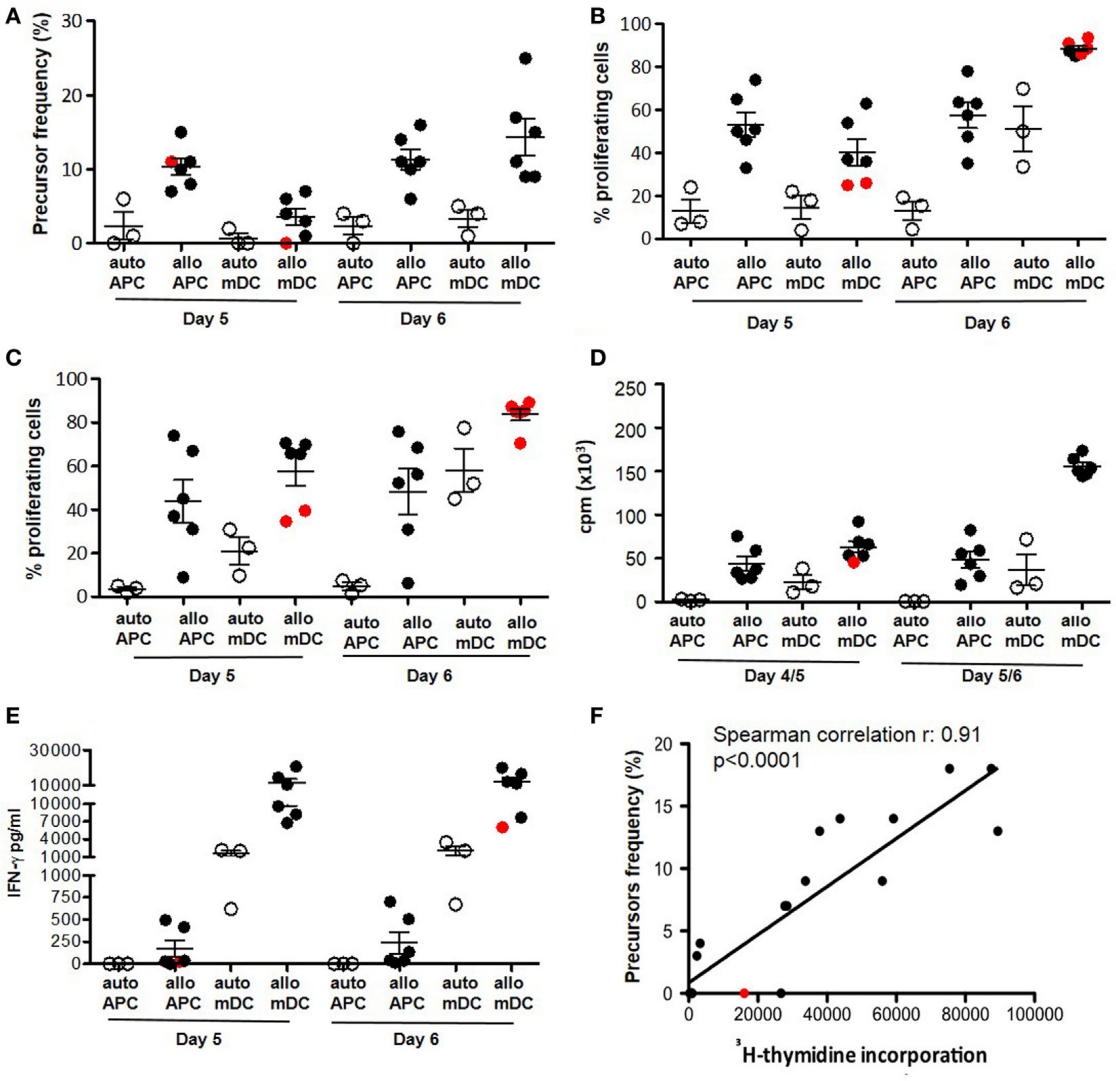
Dye-based proliferation assay to analyze alloAg-specific T-cell responses. Freshly isolated PBMCs **(A,B,D,E)** or purified CD4^+^ T lymphocytes **(C)** (from *n* = 3 healthy donors) were activated in the presence of either irradiated autologous or irradiated allogeneic CD3-depleted PBMCs (APC) (*n* = 2 allogenic stimulators) at a 1:1 responder: stimulator ratio. Alternatively, cells were stimulated with either autologous or allogenic mature DC (mDC) at a 10:1 responder:stimulator ratio for the indicated time points. The proliferative response was evaluated with proliferation dye (efluor-670; 10 µM) **(A–C,F)** (3), H-thymidine incorporation **(D,F)**, or IFN-γ secretion **(E)**. Mean ± SEM of precursor frequencies in the starting population (gated on CD3^+^ T lymphocytes) **(A)**, means ± SEM of the percentage of proliferating CD3^+^ T cells **(B,C)**, means ± SEM of cpm **(D)**, mean ± SEM of IFN-γ concentration **(E)** are plotted. Each filled dot represents an independent responder-stimulator mismatch. Open dots represent responder-stimulator autologous controls **(C)**. Cutoff for positive response was set as stimulation index (SI) vs. matched autologous stimulators >2. Red dots indicate responder-stimulator mismatch with SI <2 (non-responders). **(F)** Correlation between detection of alloAg-specific response by ^3^H-thymidine incorporation and dye-based proliferation was evaluated by Spearman’s rank correlation analysis (non-parametric). The plots show cpm at day 4/5 vs. precursor frequency of CD3^+^ T cells detected at day 6. Each dot represents an independent responder-stimulator (CD3-depleted PBMCs) match (including both auto- and allo-stimulators and both PBMC and purified CD4^+^ T cells as responders) (*n* = 18 independent determinations for cells derived from three healthy donors). The line represents the linear regression; coefficients and p values of the correlation are reported in the graphs. APC: antigen-presenting cells; mDC: monocyte-derived mature (LPS activated) dendritic cell; cpm: counts per minute; allo: allogeneic; auto: autologous.

**Figure 6:**
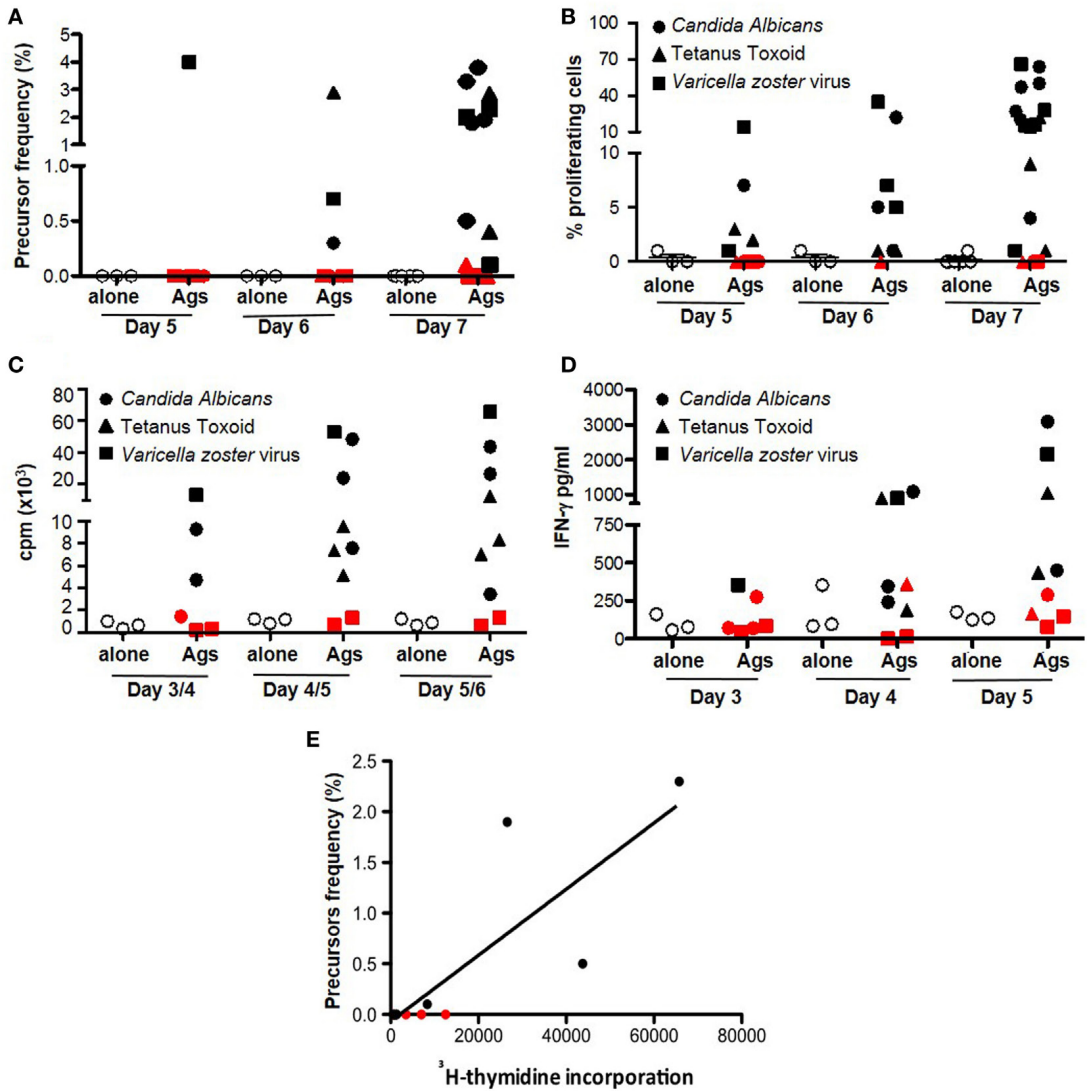
Dye-based proliferation assay to detect pathogen-derived Ag-specific T cells. Freshly isolated PBMCs were left inactivated (white circles, alone) or stimulated with *Candida albicans* spores (solid circles) or tetanus toxoid (triangles), or in the presence of total protein extract from a cell line infected with *Varicella zoster* Virus (squares) for the indicated time points. The proliferative response was evaluated by proliferation dye (efluor-670, 10 µM) of CD4^+^ T cells **(A)** and as frequency of precursors **(B)** (3), H-thymidine incorporation **(C)**, or IFN-γ secretion **(D)**. Each dot represents PBMC unstimulated or stimulated with a nominal Ag. Cutoff for positive response was set as stimulation index (SI) vs. autologous non-stimulated cells >2. Red symbols indicate donors with SI <2 (non-responders). Ag: antigen; cpm: counts per minute. Cells from ≥3 healthy donors were tested for each time point. **(E)** Correlation between detection of pathogen-specific Ag-specific response by ^3^H-thymidine incorporation and dye-based proliferation was evaluated by Spearman’s rank correlation analysis (non-parametric). The plots show cpm at day 5/6 vs. precursor frequency of proliferating CD3^+^ T cells at day 7. Each dot represents an independent responder PBMC unstimulated or stimulated with pathogen-specific Ag (*C. albicans* or tetanus toxoid or *V. zoster* Virus) (12 independent experiments were performed with cells derived from three healthy donors). The lines represent the linear regression; coefficients and *p* values of the correlation are reported in the graphs.

We evaluated the sensitivity of dye-based assays in human samples, with unknown frequencies of responder cells in comparison with ^3^H-thymidine incorporation and IFN-γ release. We evaluated the cellular response to alloAgs and pathogen-derived exogenous Ags (*C. albicans*, tetanus toxoid, and *V. zoster* Virus) (Figures 5 and 6, respectively). For alloAg-specific T-cell responses, a comparison of alloAg-specific proliferation induced by allo CD3-depleted PBMCs (allo APC) or allo monocyte-derived mDCs (allo mDC) is shown (Figure 5). As negative control, auto APC or mDC (auto mDC) was used. In this example, the allo proliferative response induced by allo APC was equally good as that induced by allo mDC. CD3-depleted PBMCs may be considered as preferred stimulator source, as their generation is much less laborious and costly than *in vitro* generated dendritic cell (DC) from allo monocytes, and tend to induce less auto background proliferation than allo mDC (Figures 5A,B). Comparison of total PBMCs and isolated CD4^+^ T cells as source of responder cells (Figures 5B,C, respectively) showed that total PBMCs may be the preferred choice, since the proliferative response was comparable to that of purified CD4^+^ T cells, and they are easier and less expensive to obtain. Of note, in case of very low expected frequencies of Ag-specific T cells, purification of the CD4^+^ T-cell pool may be advisable to increase the relative frequency of the specific T cells in culture (Figure 5C). In the case of pathogen-derived Ags, precursors’ frequencies were very low (Figure 6) and separate division peaks were not visible (Figure S2 in Supplementary Material); the precursors’ frequency calculation relied on the software peak prediction.

As expected, proliferation in response to alloAgs or pathogen-derived exogenous Ags can also be detected by ^3^H-thymidine incorporation (Figures 5D and 6C) or IFN-γ release (Figures 5E and 6D), although these two read outs did not allow to evaluate the proliferation or cytokine secretion specifically by CD4^+^ or CD8^+^ T cells within PBMCs. Interestingly, while the proliferation dye dilution and ^3^H-thymidine incorporation correlated well both in the case of alloAg-specific T-cell responses and the responses to pathogen-derived Ags (Figures 5F and 6E), correlation between the proliferation dye dilution and IFN-γ release was less evident in the case of alloAg-specific T-cell responses but present for pathogen-derived Ag-specific responses (Figure S3 in Supplementary Material). From the above experiments, it can be concluded that dye proliferation assay is suitable to detect T cell specific for alloAgs or pathogen-derived exogenous Ags. For optimal readout of alloAg responses 4–5 days of stimulation is suitable (Figure 5), while for pathogen-derived exogenous Ag responses 7 days of culture is required (Figure 6). The assay time is longer compared to other techniques (i.e., H-thymidine incorporation or cytokine profiles) (3). This time is mandatory for small population of Ag-specific T cells to reach numbers detectable and quantifiable with dye-based assays (4, 36).

The comparison of proliferation data obtained with dye-based proliferation assay and ^3^H-thymidine incorporation for alloAg- and nominal Ag-specific T-cell response gave good concordance. These results are in line with previous reports (3, 37–39). However, a less correlation was observed between proliferation dye dilution and IFN-γ production, specifically in allo mixed lymphocyte reaction. This result may be related to NK-cell activation when total allo APC is used as stimulatory cells. To avoid this possibility, the use of monocye-derived DCs would be recommended. Moreover, it has to be considered that in the proposed examples as well as in the present study, correlation is observed when high frequency of Ag-specific T cells is present in the peripheral blood and strong antigenic responses are analyzed. This is less evident when Ag-specific T cells are less frequent, as in patients with autoimmune disorders, or a less immunogenic response is studied (40).

**Figure 7:**
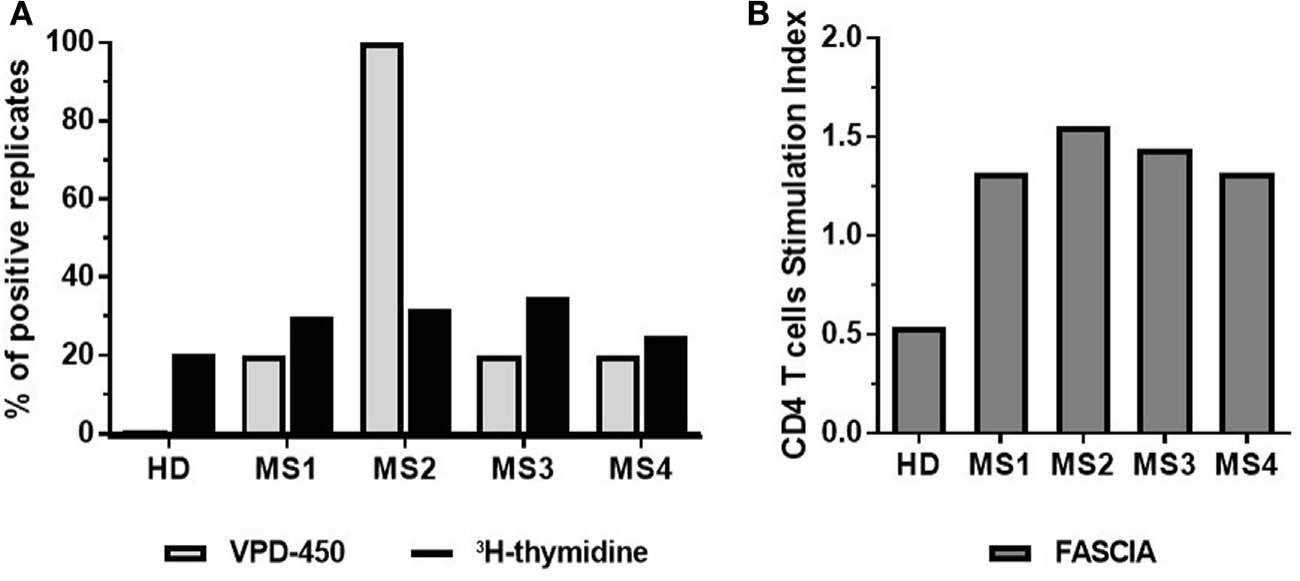
Dye-based proliferation assay to detect autoAg-specific T cells in multiple sclerosis (MS) patients. Freshly isolated PBMC **(A)** or whole blood **(B)** from four MS patients were stimulated with a mix of seven myelin peptides [myelin oligodendrocyte glycoprotein (MOG) 1–20, MOG35–55, PLP139–154, myelin basic protein (MBP) 13–32, MBP83–99, MBP111–129, and MBP146–170] for 7 days. The proliferative response was evaluated by dye-based proliferation (VPD-450, 1 µM) or ^3^H-thymidine incorporation **(A)**, or flow cytometric assay of specific cell-mediated immune response in activated whole blood (FASCIA) **(B)**. A total of 5 wells (VPD-450) or 60 wells (^3^H-thymidine) were analyzed. The % of autoAg-reactive wells/replicates (wells showing increased ^3^H-thymidine incorporation compared to the mean of non-stimulated wells) (black bars) and the % of autoAg-reactive wells/replicates from VPD-450 dilution assay (wells exhibiting a SI = stimulated wells/non-stimulated wells ≥1.5) (gray bars) are shown **(A)**. The number of CD4^+^ blast cells was determined by FASCIA **(B)**.

Detection of reactive T cells against autoAgs requires highly sensitive techniques due to the low frequency of these auto-reactive T cells in peripheral blood (26, 27). For this reason, as indicated above high numbers of cells (i.e., 1.5 × 10^5^ PBMCs/well) in several replicates should be seeded. To test the sensitivity of the proliferation dye assay in the detection of autoAg-specific T-cell responses, we tested the response of PBMC from multiple sclerosis (MS) patients to a mix of seven myelin peptides by comparing the proliferation dye (VPD-450), with ^3^H-thymidine incorporation and FASCIA. As depicted in Figure 7A (3), _3_H-thymidine incorporation is highly sensitive for detecting autoAg-specific T cells, since all patients tested exhibited increased proliferation ≥25% of analyzed wells compared to the mean of non-stimulated controls (Figure 7A). Analysis of proliferation dye dilution indicated that separate division peaks were not visible upon autoAg-specific stimulation, making it impossible to calculate PF, even with the aid of the software peak prediction program. Therefore, as alternative the frequency of autoAg-specific T cells was calculated as the percentage of positive wells defined by considering replicates showing ≥1.5 stimulation index (SI, % proliferating cells stimulated/% proliferating cells non-stimulated). Compared to ^3^H-thymidine incorporation, the VPD-450 dilution assay generated a similar% of positive replicates (≥20% positive wells) in three out of four MS patients, while for one patient all replicates were positive in the dye-based assay. In parallel, FASCIA was also performed by stimulating fresh whole blood from the same MS patients with the mix of myelin peptides. The analysis of CD4^+^ blast cells showed a SI ≥1.3 in all samples (Figure 7B), like the SI ≥1.5 detected in positive auto-reactive wells using VPD-450 dilution assay. These examples indicate that dye-based proliferation assay is sensitive enough to detect T cells specific for a given Ag, including blood samples from patients with autoimmune disease in whom the frequency of autoAg-specific T cells in peripheral blood is generally low.

The latter results are compliant with those of Zafranskaya et al. (41), who compared ^3^H-thymidine incorporation and CFSE-based assay for assessing MOG-reactive T cells in healthy donors, untreated MS patients and IFN-β-treated patients. Remarkably, data from MS patients contrast with those obtained in type 1 diabetes (T1D) patients. It was demonstrated that CFSE-based proliferation assay was more sensitive than ^3^H-thymidine incorporation to study auto-Ag-specific reactivity in T1D patients (3). In this study, all tested patients had a detectable response to glutamic acid decarboxylase (GAD), an autoAg in T1D, with CSFE dilution assay, that was revealed only in half of the patients by ^3^H-thymidine incorporation. Moreover, Segovia-Gamboa et al. (42) detected GAD- and insulin-specific responses with a CFSE-based assay using auto DCs loaded with Ag as stimulators for memory CD4^+^ T cells. We cannot exclude that the discrepancy between analyses performed in T1D patients and our data depends on the frequency of auto-reactive T cells in these patients, duration, and stage of the disease, or the immunogenicity of the autoAg. Data obtained with FASCIA assays are promising, although the assay is somewhat less sensitive than dye proliferation assay in detecting auto-reactive CD4^+^ T cells. Nevertheless, it can be an alternative in cases PBMC isolation maybe complicated as, for instance, in pediatric patients. Although analysis of autoAg-specific responses is challenging, we believe that dye-based proliferation assays represent a good choice for the enumeration of autoAg-specific T cells, since they allow for measurement of additional phenotypical and cell function related parameters critical for a better description of auto-reactive T cells and their activation.

In accordance with previous results reported using CFSE-based assay (43, 44), we showed that dye-based proliferation assay is suitable to detect autoAg-specific T cells in peripheral blood of MS patients. Moreover, the sensitivity of dye-based proliferation assay is comparable to that of ^3^H-thymidine incorporation in detecting auto-reactive T cells in MS patient’s PBMC.

## Concluding Remarks

Dye-based proliferation assays, in contrast to other approaches, offer the possibility to retrieve additional information additional to the overall proliferative response. First, the frequency of Ag-specific T-cell precursors in the starting population can be determined, which is not the case, for instance, for proliferation analyzed by ^3^H-thymidine incorporation. Furthermore, dye-based proliferation assays provide insights in the dynamics of proliferation and phenotype of the cells at different stages of proliferation within a PBMC culture. Tolerizing immunotherapy can induce Ag-specific tolerance *via* several mechanisms: (allo)Ag-specific T cells can be deleted or become anergic, and this will lower the PF (45, 46). Alternatively, the tolerizing therapy may cause the (allo)Ag-specific T cells to respond to a lower extent, leading to a restraint on cell division, while not affecting PFs. In conclusion, tracking Ag-specific T-cell responses with dye dilution represents a valuable tool to monitor tolerance induction in human. Strict attention to setup and validation of the culture conditions should be given before execution of the study while taking into consideration the disease and the type of Ag under assessment. We believe that this is the first step to harmonize the monitoring of tolerance induction, which will enable the comparison of immunological mechanisms responsible for the clinical outcomes of different tolerance-inducing studies. In addition, a well-designed and validated dye proliferation assay can be applied to other therapies aimed at increasing Ag-specific T-cell responses such as vaccination and cancer immunotherapy.

## Ethics Statement

Human peripheral blood was obtained from healthy donors upon informed consent and approval by local ethical committee (Sanquin Amsterdam, Medical University of Gdańsk, San Raffaele Scientific Institute) and in line with the Declaration of Helsinki. Human peripheral blood was obtained from four MS patients from the Multiple sclerosis Unit, Germans Trias I Pujol University Hospital (Badalona, Spain) upon informed consent in accordance with local ethical committee approval and with the Declaration of Helsinki. No patient had clinical exacerbations or was receiving corticosteroid or disease modifying treatments at the moment of the sample collection. Studies with mice were approved and in accordance with guidelines from King’s College London, UK.

## Author Contributions

AB, NM-T, SH, MF, EM-C, and SG wrote the article. AB, NM-T, AT, EM-C, and SG designed and planned experiments. NM-T, MM, AT, KP, DI-G, LP, GL, JP-O, and MF performed and analyzed experiments.

## Conflict of Interest Statement

The authors declare that the research was conducted in the absence of any commercial or financial relationships that could be construed as a potential conflict of interest.

## Abbreviations

A FACTT, a European network action to focus and accelerate cell-based tolerance-inducing therapies; Ag, antigen; allo, allogeneic; APC, antigen-presenting cell; auto, autologous; CFSE, carboxyfluorescein diacetate succinimidyl ester; cpm, counts per minute; COST, European Cooperation in Science and Technology; CTV, cell trace violet; DC, dendritic cell; DI, division index; ELISA, enzyme-linked immunosorbent assay; ELISpot, enzyme-linked immunospot; FASCIA, flow cytometric assay of specific cell-mediated immune response in activated whole blood; FBS, fetal bovine serum; GAD, glutamic acid decarboxylase; HS, human serum; OVA, ovalbumin; mAb, monoclonal antibody; MBP, myelin basic protein; MOG, myelin oligodendrocyte glycoprotein; MS, multiple sclerosis; PBMCs, peripheral blood mononuclear cells; Pen/strep, penicillin-streptomycin; PF, precursor frequency; PI, proliferation index; PLP, proteolipid protein; SEB, Staphylococcal enterotoxin B; SI, stimulation index; Tregs, regulatory T cells; tolAPCs, tolerogenic antigen-presenting cells; tolDC, tolerogenic dendritic cells; T1D, type 1 diabetes; VPD-450, violet proliferation dye 450.
