# Corrigendum: Monitoring T-Cell Responses in Translational Studies: Optimization of Dye-Based Proliferation Assay for Evaluation of Antigen-Specific Responses

**DOI:** 10.3389/fimmu.2018.00343

**Published:** 2018-02-22

**Authors:** Anja Ten Brinke, Natalia Marek-Trzonkowska, Maria J. Mansilla, Annelies W. Turksma, Karolina Piekarska, Dorota Iwaszkiewicz-Grześ, Laura Passerini, Grazia Locafaro, Joan Puñet-Ortiz, S. Marieke van Ham, Maria P. Hernandez-Fuentes, Eva M. Martínez-Cáceres, Silvia Gregori

**Affiliations:** ^1^Department of Immunopathology, Sanquin Research, Amsterdam, Netherlands; ^2^Landsteiner Laboratory, Academic Medical Centre, University of Amsterdam, Amsterdam, Netherlands; ^3^Laboratory of Immunoregulation and Cellular Therapies, Department of Family Medicine, Medical University of Gdańsk, Gdańsk, Poland; ^4^Immunology Division, Department of Cellular Biology, Germans Trias i Pujol University Hospital and Research Institute, Physiology, and Immunology, Universitat Autònoma Barcelona, Barcelona, Spain; ^5^Department of Clinical Immunology and Transplantology, Medical University of Gdańsk, Gdańsk, Poland; ^6^San Raffaele Telethon Institute for Gene Therapy (SR-Tiget), Division of Regenerative Medicine, Stem Cells and Gene Therapy, IRCCS San Raffaele Scientific Institute, Milan, Italy; ^7^MRC Centre for Transplantation, King’s College London, London, United Kingdom

**Keywords:** tolerance, monitoring, proliferation, antigen-specific, T cells, transplantation, autoimmune diseases, immune-therapies

In the original article, we neglected to include the funder National Centre for Research and Development, LIDER/160/L-6/14/NCBR/2015 to Dorota Iwaszkiewicz-Grześ. The authors apologize for this error and state that this does not change the scientific conclusions of the article in any way.

The original article has been updated.

## Conflict of Interest Statement

The authors declare that the research was conducted in the absence of any commercial or financial relationships that could be construed as a potential conflict of interest.

