# Supplementary material for: Monitoring T-Cell Responses in Translational Studies: Optimization of Dye-Based Proliferation Assay for Evaluation of Antigen-Specific Responses
Source: Front Immunol. 2017 Dec 21;8:1870. doi: 10.3389/fimmu.2017.01870 (PMC5742609; doi:10.3389/fimmu.2017.01870)
Supplement: Supplementary file 1 [file Data_Sheet_1.pdf]

Supplementary Materials:

Figure S1

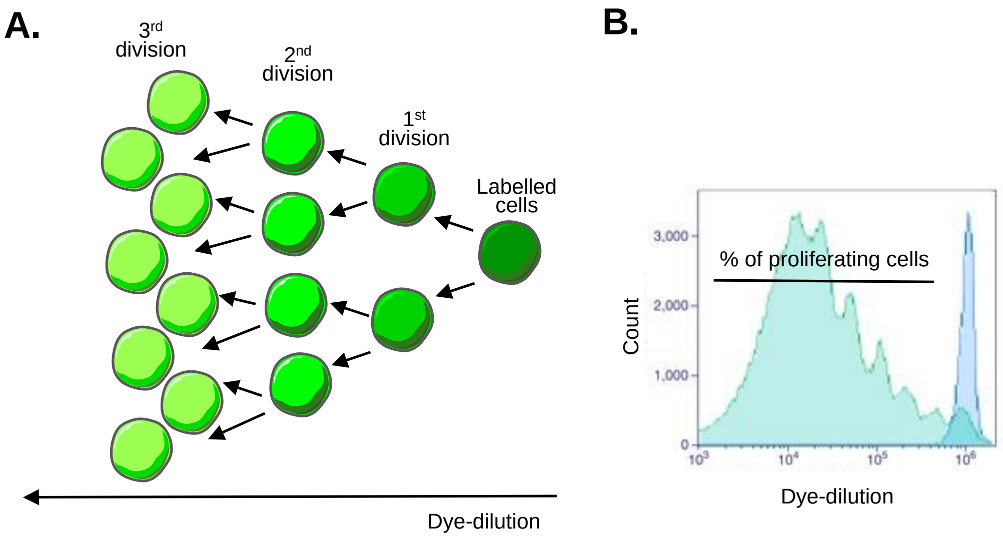

Fig. S1 Flow Cytometric Analysis of Cell Division by Dye Dilution. (A) The cell-permeant fluorescein-based dye covalently attaches to cytoplasmic components of cells, resulting in uniform bright fluorescence. Upon cell division, the dye is distributed equally between daughter cells, allowing the resolution of up to several cycles of cell division by flow cytometry. (B) Example of proliferation dye dilution on the right: blue histogram indicates labelled and unstimulated and cultured cells, green histogram indicates labelled and stimulated cells. Six peaks of cell division are visible. Line indicates the percentage of cells showing dye-dilution (% of proliferating cells).

Figure S2

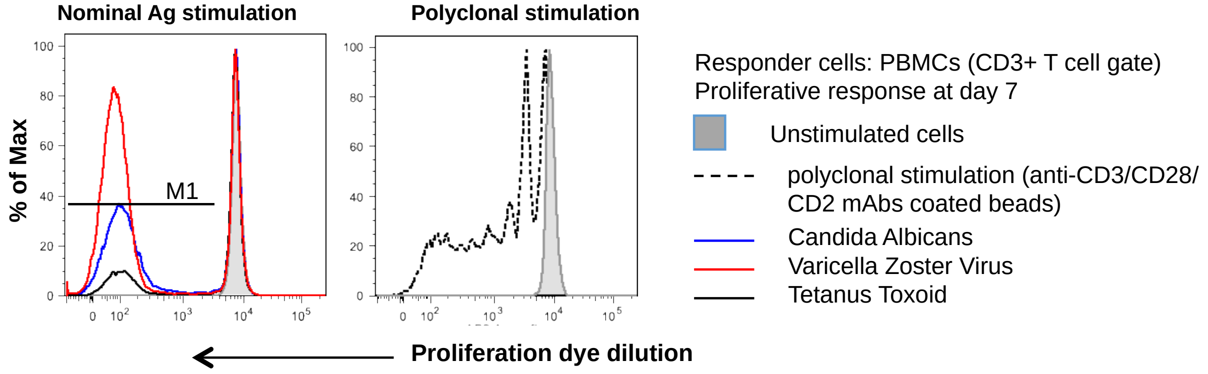

Fig. S2: Dye-based proliferation to detect nominal pathogen-specific Ag-specific stimulation: peaks are barely detectable. PBMCs were left inactivated (grey filled histogram) or stimulated with *Candida albicans* spores (blue), Tetanus Toxoid (black), or a total protein extract from a cell line infected with *Varicella zoster* Virus (red) or stimulated with a polyclonal stimulus in the form of anti-CD3/CD28/CD2 mAbs coated beads (dotted line). The proliferative response was evaluated by proliferation dye (efluor-670) dilution assay. Representative histograms are shown, depicting Ag-specific or polyclonal proliferation of PBMCs gated on alive $CD3^+$ T cells.

Figure S3

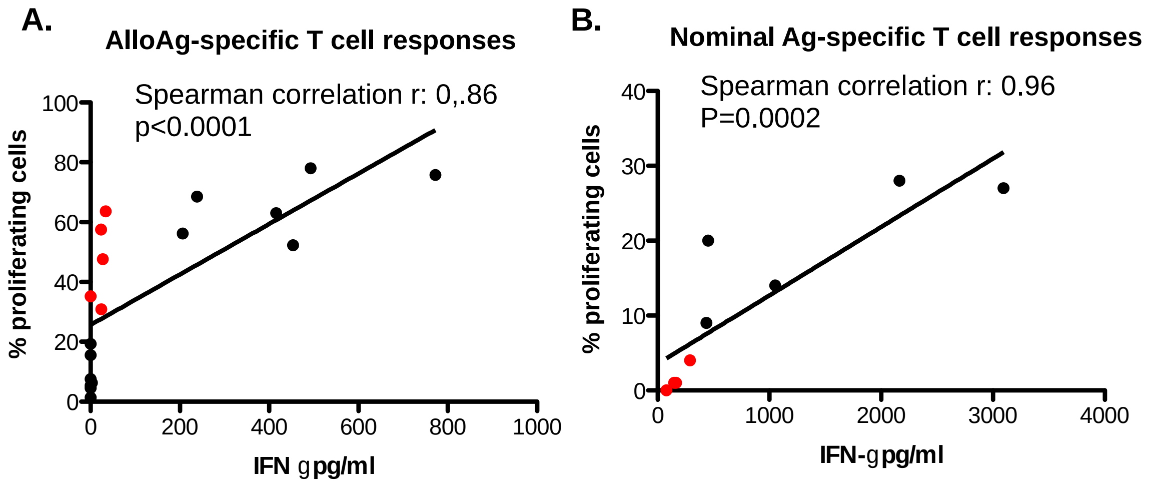

Fig. S3: Dye-based proliferation does not correlate with IFN-γ release. Correlation between detection of allo-Ag- (A) or pathogen-specific Ag- (B) specific response by proliferation dye-dilution assay and IFN-γ production was evaluated by Spearman's rank correlation analysis (non-parametric). The plots show percentage of proliferated $CD3^+$ T cells, and IFN-γ pg/ml. (A) Each dot represents an independent responder-stimulator (PBMC-CD3 depleted) match (including both auto- and allo-stimulators and both PBMC and purified $CD4^+$ T cells as responders) (n=18 independent determination using n=3 healthy donors). (B) Each dot represents an independent responder PBMC unstimulated or stimulated with pathogen-specific Ag (either *Candida albicans*, or Tetanus Toxoid, or *Varicella zoster* Virus) (n=12 independent determination using n=3 healthy donors). The lines represent the linear regression; coefficients and p values of the correlation are reported in the graphs. Red dots highlight donors for whom the two assays yield highly deviant results (non-responders according to the parameter plotted on the y-axis, responders following dye proliferation).

Table S1: Calculation of proliferation index (PI)

Day 4

N	No. events	2^i	No. Events/2^i	(No. Events/2^i)*N	
0	4086	1	4086.00		
1	2800	2	1400.00	1400.00	
2	2991	4	747.75	1495.50	
3	2897	8	362.13	1086.38	
4	633	16	39.56	158.25	
5	52	32	1.63	8.13	
6	22	64	0.34	2.06	
**total**	9395.00		2551.41	4150.31	**1.63**

Day 5

N	No. events	2^i	No. Events/2^i	(No. Events/N^i)*N	
0	8461	1	8461.00		
1	3492	2	1746.00	1746.00	
2	4090	4	1022.50	2045.00	
3	4455	8	556.88	1670.63	
4	4885	16	305.31	1221.25	
5	3428	32	107.13	535.63	
6	721	64	11.27	67.59	
total	21071.00		3749.08	7286.09	**1.94**

N is the division number, ranging from N0 to N6 in this example. No. of events in each division peak is entered (including undivided cells N0). 2i indicate the number of cells/division. To calculate proliferation index: 1. the no. of events per division peak is divided by 2^i; 2. result then multiplied by N; 3. divide the total (No. of events/N^i)*N by the total No. events.

Table S2: Calculation of precursor frequency

Day 4

N	No. events	2^i	No. events/ 2^i	% prog
0	4086	1	4086.00	61.56
1	2800	2	1400.00	21.09
2	2991	4	747.75	11.27
3	2897	8	362.13	5.46
4	633	16	39.56	0.60
5	52	32	1.63	0.02
6	22	64	0.34	0.01
total	9395		6637.41	**38.44**

Day 5

N	No. events	2^i	No. events/ 2^i	% prog
0	8461	1	8461.00	69.30
1	3492	2	1746.00	14.30
2	4090	4	1022.50	8.37
3	4455	8	556.88	4.56
4	4885	16	305.31	2.50
5	3428	32	107.13	0.88
6	721	64	11.27	0.09
total	21071		12210.08	**30.70**

N is the division number, ranging from N0 to N6 in this example. No. of events in each division peak (including undivided N0). 2^i indicate the number of cells/division. To calculate percentage of precursor cells: 1. no. of cells per division peak is divided by 2i; 2. no. of events/2^i is multiplied by 100% and divided by the total No. of events/2^i; 3: add percentages from N1 to N6.
